# THE CAREGIVING BURDEN OF CHINESE OLDER ADULTS AT THE END OF LIFE: THE ROLE OF LIVING ARRANGEMENT AND COVID-19 PANDEMIC

**DOI:** 10.1093/geroni/igae098.1746

**Published:** 2024-12-31

**Authors:** Shuo Zhang, Wenjun Zhang

**Affiliations:** Renmin University of China, Beijing, Beijing, China (People’s Republic); Renmin University of China, Beijing, Beijing, China (People’s Republic)

## Abstract

While there has been a surge of interest in studying end-of-life quality of lives of older adults in China to respond to population aging, the experiences of their caregivers remain largely unexamined. Chinese older adults in general may have to rely on their co-residing members to provide end-of-life care. To address the gap, we explored the association between living arrangement and caregiving burden during end-of-life stage before and during the COVID-19 pandemic using a representative sample. The working sample included 1043 decedents who died between 2017 and 2021 from China Longitudinal Aging Social Survey. Ordered logistic regression models were used to examine the impact of living arrangement and pandemic on caregiving burden at the end of life. Our results indicated that caregivers of older adults who lived alone and lived in empty-nest, stem, and nuclear families had higher caregiving burden than those lived in skipped-generation families. The COVID-19 pandemic increased caregiving burden and further strengthened the relationship between living arrangements and end-of-life caregiving burden. Particularly, caregivers to older adults who lived alone, lived in empty-nest, stem, and nuclear families during the pandemic had higher caregiving burden than those before the pandemic. The main findings highlight the importance of paying attention to the caregiving burden of caregivers to those who are not living in skipped generation families, especially during pandemic. Our findings implied that we need to develop comprehensive formal support system to reduce the caregiving burden for caregivers at the end of life.

